# Economic impact of Juvenile Idiopathic Arthritis: a systematic review

**DOI:** 10.1186/s12969-021-00641-y

**Published:** 2021-10-09

**Authors:** Fernando García-Rodríguez, Augusto Gamboa-Alonso, Sol Jiménez-Hernández, Lucero Ochoa-Alderete, Valeria Alejandra Barrientos-Martínez, Neri Alejandro Alvarez-Villalobos, Gabriela Andrea Luna-Ruíz, Ingris Peláez-Ballestas, Ana Victoria Villarreal-Treviño, Manuel Enrique de la O-Cavazos, Nadina Rubio-Pérez

**Affiliations:** 1 Department of Pediatrics, Hospital Universitario “Dr. José E. González”, Universidad Autónoma de Nuevo León, Madero y Gonzalitos SN, Col. Mitras Centro, C.P. 64460 Monterrey, Mexico; 2 Plataforma INVEST Medicina UANL-KER Unit Mayo Clinic (KER Unit México), Universidad Autonoma de Nuevo Leon, Monterrey, Mexico; 3 Departamento de Economía, Universidad Iberoamericana, Mexico City, Mexico; 4 Rheumatology Unit, Hospital General de México “Dr. Eduardo Liceaga”, Mexico City, Mexico

**Keywords:** Juvenile Idiopathic Arthritis, Economic impact, Direct costs, Burden, Systematic review

## Abstract

**Background:**

Juvenile Idiopathic Arthritis (JIA) requires complex care that generate elevated costs, which results in a high economic impact for the family. The aim of this systematic review was to collect and cluster the information currently available on healthcare costs associated with JIA after the introduction of biological therapies.

**Methods:**

We comprehensively searched in MEDLINE, EMBASE, Web of Science, Scopus, and Cochrane Databases for studies from January 2000 to March 2021. Reviewers working independently and in duplicate appraised the quality and included primary studies that report total, direct and/or indirect costs related to JIA for at least one year. The costs were converted to United States dollars and an inflationary adjustment was made.

**Results:**

We found 18 eligible studies including data from 6,540 patients. Total costs were reported in 10 articles, ranging from $310 USD to $44,832 USD annually. Direct costs were reported in 16 articles ($193 USD to $32,446 USD), showing a proportion of 55 to 98 % of total costs. Those costs were mostly related to medications and medical appointments. Six studies reported indirect costs ($117 USD to $12,385 USD). Four studies reported costs according to JIA category observing the highest in polyarticular JIA. Total and direct costs increased up to three times after biological therapy initiation. A high risk of reporting bias and inconsistency of the methodology used were found.

**Conclusion:**

The costs of JIA are substantial, and the highest are derived from medication and medical appointments. Indirect costs of JIA are underrepresented in costs analysis.

**Supplementary Information:**

The online version contains supplementary material available at 10.1186/s12969-021-00641-y.

## Background

Juvenile Idiopathic Arthritis (JIA) is a generic term, used to describe a group of heterogeneous diseases characterized by chronic arthritis with onset before the age of 16 [1]. JIA is the most common chronic rheumatic disease during childhood with an worldwide incidence between 1.6 and 23 per 100,000 children, which varies according to the region and subtype of the disease [2].

Patients with JIA have symptoms of joint inflammation, morning stiffness, pain, contractures, fatigue, abnormal growth, and functional limitation [3]. The differences between subtypes of JIA are the number of joints with arthritis, severity of the disease and extraarticular symptoms, however, they all are considered as chronic illnesses with a long-term treatment and follow up [4].

The treatment of JIA must be multilevel, with a pediatric rheumatologist, psychological support, physical therapy, nutrition, and family support. Within pharmacological treatment, a wide range of options with different costs and effectiveness could be found. Some studies have shown that the costs increase in relation to disease activity and progression to disability [5]. With this perspective, considering the multiple medical appointments, laboratory tests, medications, and indirect costs, this disease generates elevated costs, which results in a high economic impact for the family [6].

The costs can be dived in those directly related with healthcare services (direct healthcare costs), and those not related to healthcare services (indirect healthcare costs) [7]. Therefore, the economic burden for the family depends on factors such as healthcare coverage available, income status, actual treatment, disability, and the intervention needed [8].

There have been some reports about the costs of illness for JIA, but its variability is important according to the region, social context, and healthcare system. The aim of this systematic review was to collect, and cluster the information currently available on healthcare costs associated with JIA in the world after the introduction of biological therapies.

## Methods

### Study design

We performed a systematic review to estimate the direct and indirect costs in patients with JIA, regardless of the subtype or region. This report followed a rigorous systematic review protocol that adhered to the Preferred Reporting Items for Systematic Reviews and Meta-Analyzes (PRISMA) [9] recommendations and was registered in PROSPERO (https://www.crd.york.ac.uk/prospero) with the code CRD42019135865.

### Eligibility criteria

We included complete economic evaluations (cost-effectiveness, cost-utility, cost-benefit, cost minimization and cost-consequences analysis), partial economic evaluations (cost analysis, cost description and cost-outcome), and individual studies with cost reporting (clinical trials and observational studies) regardless of publication status, size, or language. Primary studies published after 2000 that report total, direct and/or indirect costs related to JIA for at least one year were included, since the aim was to analyze the costs after the introduction of biological therapies. Non-primary studies (narrative or systematic reviews, letters to the editor, comments, and editorials), studies published in a non-peer-reviewed source (conference proceedings, thesis repositories, non-scientific journals, non-peer-reviewed journals, and books), and studies where information to determine eligibility was not available, were excluded.

### Study identification

A comprehensive search was carried out by an experienced librarian, advised by the principal investigators. The databases consulted were Ovid MEDLINE, EMBASE, Web of Science, Scopus, Cochrane Database of Systematic Reviews and Cochrane Central Register of Controlled Trials for studies from January 1, 2000 to the date of the search (July 27, 2019). A search update was performed on March 11, 2021. The search strategy is available as Supplementary material. Additional references were searched looking at narrative review references and consulted with experts.

### Selection of studies

Studies were entered into systematic review software (DistillerST, Ottawa, Canada). To ensure the reliability of selection among investigators, a pilot test was performed with a random sample of 60 studies derived from the search; these were reviewed for inclusion criteria by means of title and abstract. The exercise was repeated until we achieved a kappa 0.7 between the reviewers.

Reviewers (two pediatric rheumatologists, one fellow in Pediatric Rheumatology, and three medical students with experience on systematic reviews) worked independently and in duplicate to evaluate titles and abstracts on the selection criteria. After abstract screening and retrieval of potentially eligible studies, full-text publications were assessed for eligibility, with adequate inter-reviewer agreement (kappa 0.61). Duplicate studies and studies with overlapping populations were excluded. Disagreements were reviewed by a third reviewer and their inclusion was subsequently decided by consensus.

### Data collection and management

Independently and in duplicate using a standardized database, the reviewers collected the following information from eligible studies: (1) study general data (author, year of publication, title, country, region, study design, follow-up time and currency), (2) participants characteristics (sample number, type of JIA), (3) total costs (TC), (4) direct costs (DC), and (5) indirect costs (IC).

The DC included those derived from medical appointments, medications (DMARD, biologics, NSAID, steroids, intra-articular injections, prophylaxis/supplements), laboratory tests, clinical imaging, surgeries, hospitalizations, physiotherapy, devices, alternative medicine, administration of medications, adverse events, and complications. Also included derivatives of transportation, home adequacy, caregiver accommodation, travel expenses, informal and formal patient care, and insurance payments.

In the IC, those related to the loss of productivity of patients and caregivers were considered (through missed school days or educational support to the patient, absenteeism from work of the patient/caregiver, general work impact of the patient/caregiver, early retirement of the patient/caregiver, and the estimated costs of caregiver productivity).

The costs were converted to United States dollars (USD) using the OANDA’s currency calculator tool (https://www1.oanda.com/lang/es/currency/converter/) considering the data collection date of the study. An inflationary adjustment was made as of December 31, 2019, with the Inflation Tool 2020 (https://www.inflationtool.com/).

### Risk of bias in individual studies and quality assessment

The risk of bias of the economic evaluations was measured using the Quality of Health Economic Studies instrument [10]. Other types of individual studies reporting costs were assessed using the Version 2 of the Cochrane Risk of Bias Tool [11], NIH Quality Assessment Tool for Observational Cohort and Cross-Sectional Studies [12], and the Newcastle-Ottawa Quality Assessment Form for Cohort Studies [13] according to their design. Reviewers worked independently and in duplicate to assess risk of bias. Disagreements were resolved by consensus.

## Results

### Selection of studies

**Fig. 1:**
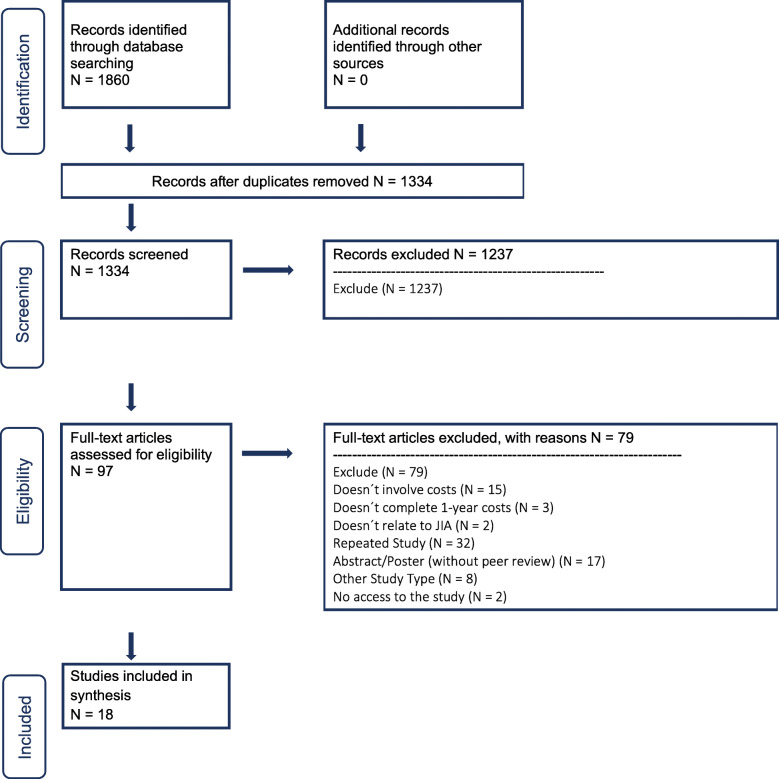
Process of study selection

A total of 1,334 studies were obtained through the systematic search, of which 18 were finally eligible after the selection process (Fig. 1). Eleven were economic studies [1, 7, 14–22], three retrospective observational [23–25] and four cohorts [4, 26–28].

### Characteristics of studies

The articles originate from Europe (12, 66.7 %), North America (4, 22.2 %), one included population from those two regions (5.5 %), and one from Asia (5.5 %). The countries included in the studies were the United Kingdom, Germany, Finland, Canada, United States of America (USA), Turkey, Italy, the Netherlands, Sweden, France, Bulgaria, and India (Table 1).

**Table 1:** Characteristics of studies included

Article	Country	Currency (Year)	Design	N	Follow-up^a^	Total costs		Direct costs		Indirect costs	
						Reported	Adjusted^b^	Reported	Adjusted^b^	Reported	Adjusted^b^
Angelis, 2016 [7]	United Kingdom	EUR (2012)	Economic study	23	12	31,546	44,832	22,831	32,446	8,715	12,385
Bernatsky, 2007 [23]	Canada	CAD (2005)	Retrospective observational	155	24			3,002	3,298		
Ens, 2013 [4]	Canada	CAD (2009)	Cohort	54	12	1,063	1,122	708	748	355	375
Minden, 2004 [17]	Germany	EUR (1999)	Economic study	215	12	3,471	5,591	1,899	3,059	1,571	2,530
Haapasaari, 2004 [26]^cd^	Finland	USD (2000)	Cohort	31	12	7,732	11,753	7,339	11,155	418	635
Hughes, 2018 [14]^de^	United Kingdom	GBP (2016)	Economic study	126	18	15,980	23,117				
Yucel, 2012 [27]	Turkey	EUR (2009)	Cohort	100	12	3,994	6,844	3,913	6,705	81	139
Kuhlmann, 2016 [1]	Germany, Italy, Spain, France, United Kingdom, Bulgaria, and Sweden	EUR (2012)	Economic study	162	12^f^	30,034	42,682				
Mars, 2019 [25]	Finland	EUR (2014)	Retrospective observational	119	106			3,631	5,450		
Mars, 2019 [24]	Finland	EUR (2014)	Retrospective observational	137	106			3,155	4,736		
Marshall, 2019 [28]^g^	United States of America	USD (2016)	Cohort	3,815	12			10,175	10,830		
Minden, 2009 [18]	Germany	EUR (2018)	Economic study	369	12	4,663	5,312	4,172	4,752		
Prince, 2011 [19]^d^	Netherlands	EUR (2005)	Economic study	49	39			12,478	15,949		
Shenoi, 2018 [20]^h^	France, Germany, Netherlands, United Kingdom, and United States of America	USD (2016)	Economic study	61	12	1,130	1,204	969	1,033		
Thornton, 2008 [22]	United Kingdom	GBP (2008)	Economic study	276	12			1,649	2,709		
Khatun, 2021 [15]	India	INR (2018)	Economic study	60	12	19,463	310	12,133	193	7,350	117
Kip, 2020 [16]^i^	Netherlands	EUR (2019)	Economic study	691	120			2,103	2,436		
Thakral, 2020 [21]^j^	United States of America	USD (2014)	Economic study	97	48			3,929	4,243		

*CAD* Canada dollar, *USD* United States dollar, *EUR* Euro, *GBP* Great Britain pound, *INR* Indian rupee

^a^Expressed in months

^b^Adjusted to inflation and converted to US Dollar. Based on exchange rate on December 31, 2019

^c^This article reports quarterly costs

^d^After biologic therapy

^e^This article reports costs on a period of 18 months

^f^Extrapolated from a 6-month period

^g^Only JIA related Costs

^h^Only Systemic JIA

^i^This article reports only costs related to medication

^j^Only Oligoarticular JIA

In the 18 articles included, 6,540 patients were found. In 11 articles it was possible to obtain data on JIA classification (2,121 patients), being oligoarticular the most frequent (Supplementary Table 1). The follow-up was 12 months in 11 studies [1, 4, 7, 15, 17, 18, 20, 22, 26–28], in the rest it was variable with a maximum of 10 years [14, 16, 19, 21, 23–25] (Table 1).

### Information on JIA costs

**Fig. 2:**
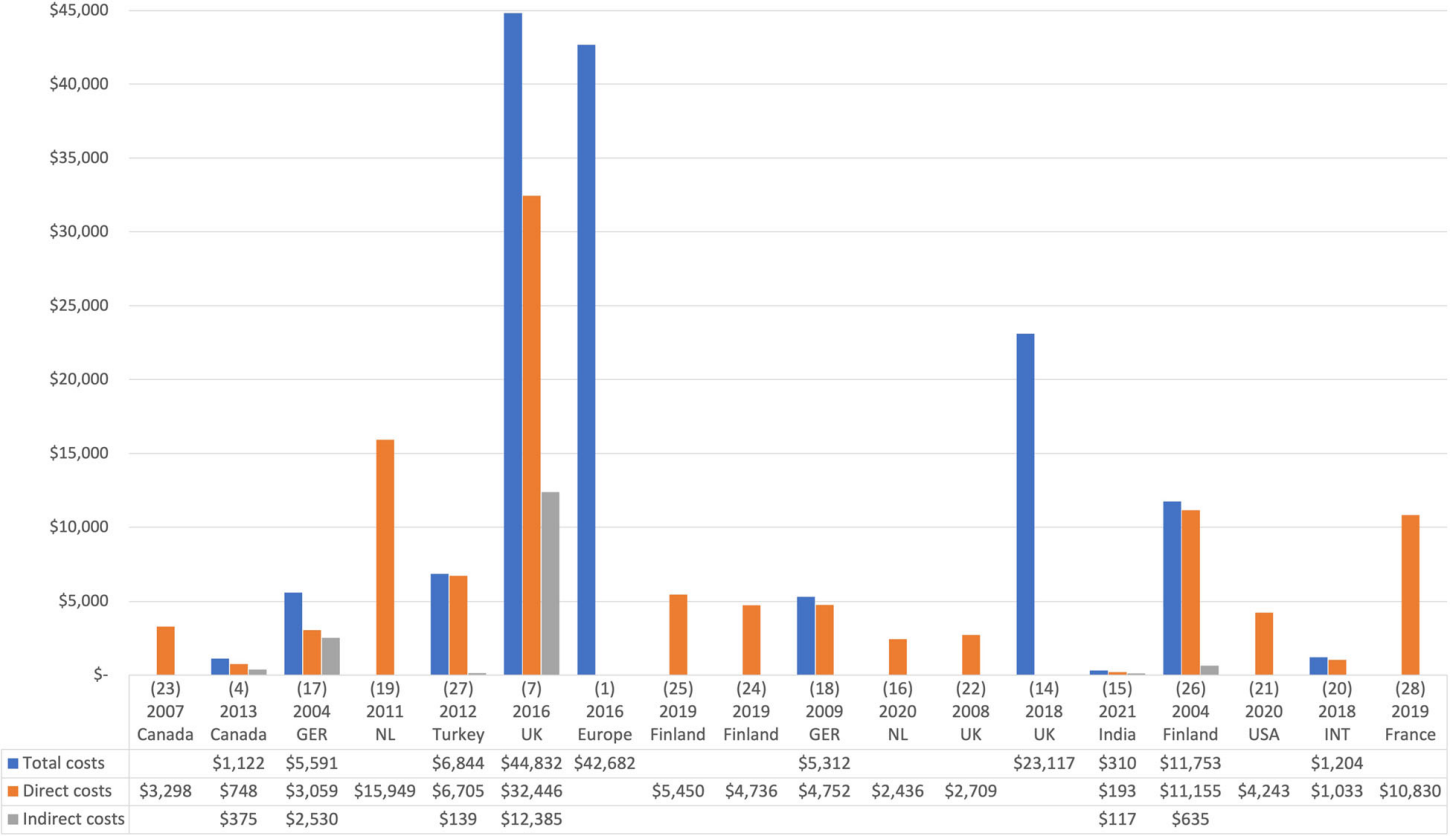
Total, direct, and indirect costs reported on eligible studies. Notes: References are shown in parenthesis above the year of publication. Costs are adjusted to inflation and converted to US Dollar. Based on exchange rate on December 31, 2019. Europe describes a study that includes patients from Germany, Italy, Spain, France, United Kingdom, Bulgaria, and Sweden. INT describes a study that includes patients from France, Germany, Netherlands, United Kingdom, and United States of America. NL: Netherlands. GER: Germany. USA: United States of America. UK: United Kingdom

We found important variability in the methodology used to calculate costs, so we were unable to perform a meta-analysis. The TC were reported in 10 articles, ranging from 310 [15] to 44,832 [7] USD per year (Fig. 2). The costs reported by studies from European countries [1, 7, 14, 17, 18, 27] were considerably higher than those reported by other regions [4, 15, 20, 26] (Table 1).

DC were reported in 16 articles, which ranged between 193 [15] and 32,446 [7] USD. Lower costs were observed in the Indian report [15] followed by those from Canada [4, 23], while costs of Europe [7, 16–19, 22, 24–27] and the USA [20, 21, 28] were variable, but consistently higher. Eight studies reported TC and DC, showing a proportion of 55 to 98 % [4, 7, 15, 17, 18, 20, 26, 27]. The highest proportion of DC was secondary to medications, hospitalizations, and medical appointments, although other items were not consistently reported (Table 2). One study reports costs on medical appointments and physiotherapy, but not a summatory of DC [14] and one reports DC but not were specific described [24]  Two studies reports DC but specific costs were described as proportions, in which the most important areas were medical appointments, medications and transportation in one (25 %, 14 %, and 11 %, respectively) [17], and medications in the other (90.4 %) [25]. One study reports costs only related to medication [16].

**Table 2:** Detailed direct health care cost description from eligible studies

Article	Original currency	Rheumatology medical appointments^a^	Other doctors medical appointments^a^	Medications^a^	Hospitalization^a^	Physiotherapy^a^	Devices^a^	Transportation^a^
Angelis, 2016 [7]	EUR 2012	5,925		9,475	1,488		162	106
Bernatsky, 2007 [23]	CAD 2005	383	447	1,435	406	99	53	
Ens, 2013 [4]	CAD 2009	345	123	189		1	51	37
Haapasaari, 2004 [26]^bc^	USD 2000			5,782		2,310		
Hughes, 2018 [14]^cd^	GBP 2016	69	30			17		
Yucel, 2012 [27]	EUR 2009	94		5,794	46	35	21	322
Marshall, 2019 [28]^e^	USD 2016	1,727	8,666	6,626	2,251			
Minden, 2009 [18]	EUR 2018			2,247	1,577	285		150
Prince, 2011 [19]^c^	EUR 2005			13,355				
Shenoi, 2018 [20]^f^	USD 2016							1,014
Thornton, 2008 [22]	GBP 2008	1,731	898	408				
Khatun, 2021 [15]	INR 2018	1.90		99	51			34
Kip, 2020 [16]	EUR 2019			2,436				
Thakral, 2020 [21]^g^	USD 2014	348		3,161				

*CAD* Canada dollar, *USD* United States dollar, *EUR* Euro, *GBP* Great Britain pound, *INR* Indian rupee

^a^Adjusted to inflation and converted to US Dollar. Based on exchange rate on December 31, 2019

^b^This article only reports quarterly cost

^c^Costs after biologic therapy started

^d^This article reports costs on an 18-months period

^e^Only JIA related Costs

^f^Only Systemic JIA

^g^Only Oligoarticular JIA

In addition six studies reported IC (117 [15] to 12,385 [7] USD) representing between 2 and 45 % of the TC [7, 15–17, 26, 27] (Fig. 2).

### Costs according to JIA subtypes

Four studies reported costs according to JIA category [18, 20, 22, 27]. One exclusively included patients with systemic JIA [20], three reported TC [18, 20, 27], and only one IC [27]. Although all reported DC, one did it in a general way, without specifying it by category [27].

Higher TC and DC were observed in patients with polyarticular JIA. The distribution of costs in the rest of the categories was variable, although lower in the studies from United Kingdom [22] and the USA [20] compared to the European ones [18, 27] (Table 3 and Supplementary Table 2). One study reports detailed costs related to medication, but other areas of DC were not included (Supplementary Table 2) [16].

**Table 3:** Total, direct, and indirect costs by category of JIA

	Total costs^a^			Direct costs^a^				Indirect costs^a^
Article	Yucel, 2012 [27]	Minden, 2009 [18]	Shenoi, 2018 [20]	Yucel, 2012 [27]	Minden, 2009 [18]	Thornton, 2008 [22]	Shenoi, 2018 [20]	Yucel, 2012 [27]
Original currency	EUR 2009	EUR 2018	USD 2016	EUR 2009	EUR 2018	GBP 2008	USD 2016	EUR 2009
All categories	6,844	5,312		6,705	4,752	2,709		139
Oligoarthritis	3,182	9,473			8,498	2,594		
Polyarthritis	10,359	18,423			15,815	3,028		
ERA	8,274	6,238			5,711	3,255		
Psoriatic	5,516	4,562			4,143	1,999		
Systemic	3,076	8,972	1,204		7,899	3,169	1,033	
Undifferentiated					5,327	2,921		

*ERA* Enthesitis-related arthritis, *USD* United States dollar, *EUR* Euro, *GBP* Great Britain pound

^a^Adjusted to inflation and converted to US Dollar. Based on exchange rate on December 31, 2019

### Costs according to JIA treatment

Four studies reported costs according to the treatment used [14, 15, 19, 29]. Two analyzed the costs before and after the start of etanercept [19, 26], one of adalimumab [14], and one compared patients with and without biologics [15]. Three considered TC, three DC, and two IC.

The TC increased in those with biological therapy, this derived from a considerable increase (up to three times more) in DC. On the other hand, IC were cut in half in one study [26] and increased three times in the other [15] (Table 4).

**Table 4:** Total, direct, and indirect costs by treatment of JIA

	Total costs^a^			Direct costs^a^			Indirect costs^a^	
Article	Haapasaari, 2004 [26]^b^	Hughes, 2018 [14]^c^	Khatun, 2021 [15]	Haapasaari, 2004 [29]^b^	Prince, 2011 [19]	Khatun, 2021 [15]	Haapasaari, 2004 [26]^b^	Khatun, 2021 [15]
Original currency	USD 2000	GBP 2016	INR 2018	USD 2000	EUR 2005	INR 2018	USD 2000	INR 2018
Without biologic therapy	10,721	9,039	200	9,552	4,753	172	1,277	28
With biologic therapy	11,753	23,117	495	11,155	15,949	409	635	86

*USD* United States dollar, *EUR* Euro, *GBP* Great Britain pound, *INR* Indian rupee

^a^Adjusted to inflation and converted to US Dollar. Date December 31, 2019

^b^This article only reports quarterly costs

^c^This article reports costs in an 18-month period

### Costs by country

Only one study reported an analysis of costs by country of origin, including patients from six European countries [1]. That study describes DC into health care costs and non-health care costs. The TC oscillated between 4,050 and 51,578 USD, with Bulgaria being the one with the lowest costs. Most of the cost was attributed to DC. Three countries did not report IC, while United Kingdom had the highest figures in this area (Supplementary Table 3).

### Risk of bias

Regarding economic studies, we found that two of eleven articles was classified as low quality due to problems in the quality of the analysis, methodology and measurement. The three cross-sectional and observational articles were classified as low risk of bias. All four cohort articles were classified as of good quality (Supplementary Tables 4, 5, and 6).

## Discussion

This study systematically reviews the information on costs in JIA in the last 20 years, additionally analyzes it differences between regions, categories, and treatments. Annual TC ranged from 1,122 to 44,832 USD, at least half of which were related to DC in the eight studies that reported both costs. Unfortunately, detailed information on DC were found in a minority of studies, reporting mostly those related with medications and medical appointments. Besides, the report of IC was vague and scarce. Similar costs were found in patients with inflammatory bowel disease [30].

On the other hand, the costs derived from JIA that we found are higher than those reported in chronic arthritis in adults. DC of JIA were up to 32,446 USD in the United Kingdom [7], 15,949 USD in the Netherlands [19], and 10,830 USD in USA [28], in contrast with those found for rheumatoid arthritis, ranged between 1,862 USD and 20,262 USD in different reports worldwide [31–35]. Furthermore, IC were 12,385 USD in the United Kingdom [7] and 2,530 USD in Germany [17] for JIA, comparable with those reported in a systematic review on ankylosing spondylitis (6,454 USD) [36].

Considering the heterogeneity of JIA, it was unexpected that costs were higher in patients with polyarticular JIA when the incidence of hospitalizations, complications and mortality are describe as higher in systemic JIA, and there are reports of more disability rates in enthesitis-related JIA [37]. This may be associated to lower remission rates in rheumatoid factor positive-polyarticular JIA, increasing the time of therapy, or due to the more frequent use of biologics to treat it [38–40], however, we were unable to carry out a more in-depth analysis in this subject.

The costs after the initiation of biological therapy increased in the studies that reported it derived from an increase in DC, similar to data from other chronic inflammatory diseases [41–43]. Despite this findings, the information collected in our review doesn’t allow us to analyze the cost-effectiveness of these therapies on JIA due to the lack of information on IC (including health-related quality of life) before and after start of biologics and the relatively short follow-up in most of the studies. These non-monetary costs have been studied in recent years and will need to be included in cost-benefit evaluations in the future [44–46].

Finally, most of the studies included presented data from Europe and USA, and the only article from a low-middle-income country shows a considerably lower cost than the rest of the reports. This could be related to lesser living expenses, lower access to comprehensive care and biological therapy, or since some expenses were covered by the government or non-governmental organizations [15]. The lack of information from other developing countries limits the generalization of the results and, therefore, the real burden of the disease.

### Implications for research

This review exhibits several gaps on this topic. First, the results show great variability between the studies, associated to the different methods to quantify costs in countries and health systems, the perspective of the chosen cost, the definition of the cost (fee, out of pocket, public price, bidding, etc.), and the ways of reporting it. The need for a consensus report in costs of JIA, especially from the patient’s perspective, is essential. The wide variability in costs reflects the low recording of the real costs of those who suffer this disease, which is reinforced by the little information related to indirect costs. Furthermore, the lack of data on absenteeism and presenteeism of patients and their caregivers is notable, as well as information regarding the long-term economic impact on these families (disability, limitation, early retirement, etc.).

Although we found some studies that addressed costs from a therapeutic perspective, only one type of biological therapy was studied, and they focused the measurement on DC. It is necessary to explore the variation in IC before and after the start of different biological therapies to establish the long-term economic benefits.

On the other hand, current literature reports costs by generalizing those across the course of JIA, thus costs at different time points during the disease journey (i.e., recent diagnosis, remission, flare, maintenance, etc.) cannot be identified. This could be an important approach to understand the most critical moments of need for financial support for those families.

Finally, if the costs and the proportion of these in relation to family income are reported, a better comparison between different regions would be achieved.

### Strengths and limitations

The extensive and rigorous search in different databases, without language restrictions and carried out by an expert medical librarian, minimizes the probability of losing information, however, it is possible that there are data on costs in sources not included in this work, such as thesis, technical reports, and conferences. The risk of reporting bias is high, particularly due to the lack of consistency in the economic aspects described, different definitions, and reports based on the perspective of health institutions and external payers. It was not possible to perform a meta-analysis of costs, which would have been of great relevance to identify the areas of greatest need for support for patients and their families. Regardless of these limitations, this review has important strengths due to the synthesis of all the available evidence following a pre-designed protocol, with reproducible judgments on the selection of studies, quality criteria and data analysis.

## Conclusions

This study synthesized the costs of JIA and highlights the financial risk that families could face during the disease trajectory. Most studies focus on total or direct costs, while indirect costs are underreported. Despite this, the information collected allows us to identify that the costs of JIA are substantial and probably the highest are derived from medication and medical appointments. Which evidences the great economic impact of JIA and how catastrophic it can be for a family.

A high risk of cost reporting bias was found and the variability of costs and the way they are measured is high, which makes it impossible to generalize the findings, although the values were higher in Europe, as well as high in polyarticular JIA.

It is necessary to standardize the reports and generate information from developing countries to obtain a more accurate analysis of the impact of the disease in the world.

## Supplementary Information

**Additional file 1. Supplementary search strategy.**

**Additional file 2. Supplementary tables.**

## Data Availability

The datasets used and/or analyzed during the current study are available from the corresponding author on reasonable request.

## Abbreviations

JIA: Juvenile Idiopathic Arthritis
PRISMA: Preferred Reporting Items for Systematic Reviews and Meta-Analyzes
TC: Total costs
DC: Direct costs
IC: Indirect costs
DMARD: Disease modifying drugs and synthetic immunosuppressants
NSAID: Non-steroidal anti-inflammatory drugs
USD: United States dollars
USA: United States of America
CAD: Canada dollar
EUR: Euro
GBP: Great Britain pound
INR: Indian rupee
