# Supplementary material for: Economic impact of Juvenile Idiopathic Arthritis: a systematic review
Source: Pediatr Rheumatol Online J. 2021 Oct 9;19:152. doi: 10.1186/s12969-021-00641-y (PMC8502332; doi:10.1186/s12969-021-00641-y)
Supplement: Supplementary file 1 — Additional file 1. Supplementary search strategy. [file 12969_2021_641_MOESM1_ESM.docx]

**Supplementary material. Search Strategy.**

**Summary July 27, 2019.**

| **Database** | **Before deduplication** | **After deduplication** |
| --- | --- | --- |
| MEDLINE | 90 | 85 |
| Embase | 653 | 651 |
| Web of Science | 266 | 124 |
| Scopus | 548 | 223 |
| CCRCT | 28 | 28 |
| CDSR | 103 | 103 |
| **TOTAL** | **1688** | **1214** |

**Examples**

| **MEDLINE** | | |
| --- | --- | --- |
| **Step** | **Type** | **Results** |
| 1 | exp juvenile rheumatoid arthritis/ or exp systemic juvenile idiopathic arthritis/ | 30363 |
| 2 | ("juvenile idiopathic arthritis" or "JIA" or "SJIA" or (Arthritis and "Juvenile Chronic") or (Arthritis and "Juvenile Enthesitis-Related") or (Arthritis and "Juvenile Idiopathic") or (Arthritis and "Juvenile Psoriatic") or (Arthritis and "Juvenile Rheumatoid") or (Arthritis and "Juvenile Systemic") or ("Chronic Arthritis" and Juvenile) or ("Enthesitis Related Arthritis" and Juvenile) or ("Enthesitis-Related Arthritis" and Juvenile) or ("Idiopathic Arthritis" and Juvenile) or "Juvenile Arthritis" or "Juvenile Chronic Arthritis" or "Juvenile Enthesitis-Related Arthritis" or "Juvenile Idiopathic Arthritis" or "Juvenile Oligoarthritis" or "Juvenile Onset Still Disease" or "Juvenile Onset Stills Disease" or "Juvenile Psoriatic Arthritis" or "Juvenile Rheumatoid Arthritis" or "Juvenile Systemic Arthritis" or "Juvenile-Onset Still Disease" or "Juvenile-Onset Still's Disease" or "Juvenile-Onset Stills Disease" or (ligoarthritis and Juvenile) or (Polyarthritis and Juvenile and "Rheumatoid Factor Negative") or (Polyarthritis and Juvenile and "Rheumatoid Factor Positive") or ("Psoriatic Arthritis" and Juvenile) or ("Rheumatoid Arthritis" and Juvenile) or ("Still Disease" and "Juvenile Onset") or ("Still Disease" and "Juvenile-Onset") or ("Still's Disease" and "Juvenile Onset") or ("Still's Disease" and "Juvenile-Onset") or ("Stills Disease" and "Juvenile-Onset") or ("Systemic Arthritis" and Juvenile)).mp. [mp=ti, ot, ab, sh, hw, kw, tx, ct, tn, dm, mf, dv, fx, dq, nm, kf, ox, px, rx, ui, sy] | 36227 |
| 3 | 1 or 2 | 38383 |
| 4 | exp "cost"/ or exp "cost of living"/ or exp "health care cost"/ | 928136 |
| 5 | (Cost or Costs or "Economic burden" or "related costs" or "direct healthcare costs" or "direct non-healthcare costs" or "loss of labor productivity" or "average annual costs" or (Costs and "Cost Analysis") or Affordabilities or Affordability or "Analyses cost" or "Cost-Minimization" or "Analysis cost" or "Cost Analyses" or "Cost Analysis" or "Cost Comparison" or "Cost Comparisons" or "Cost Measure" or "Cost Measures" or "Cost Minimization Analysis" or "Cost-Minimization Analyses" or "Cost-Minimization Analysis" or Pricing or "Cost Efficiency Analysis" or "Health Care Costs" or "Medical Care cost" or (Cost and Treatment) or (Costs and Health Care) or (Costs and Healthcare) or (Costs and "Medical Care") or (Costs and Treatment) or "Health Care Cost" or "Health Cost" or "Health Costs" or "Healthcare Cost" or "Healthcare Costs" or "Medical Care Cost" or "Medical Care Costs" or "Treatment Cost" or "Treatment Costs" or "Cost-Benefit Analysis" or "Cost Benefit" or "Cost-Benefit" or "Cost-Utility" or "Cost-Effectiveness" or "Cost-Utility" or (Benefits and Costs) or "Cost Benefit" or "Cost Effectiveness" or "Cost-Utility" or "Cost-Effectiveness" or "Cost-Utility" or "Economic Evaluation" or "Economic Evaluations" or (Evaluation and Economic) or (Evaluations and Economic) or "Health Expenditures" or "Out-of-Pocket" or "Direct Expenditure" or "Direct Expenditures" or "expenditures Indirect" or (expenditure and "Out-of Pocket") or Expenditures or (expenditures and Direct) or (Expenditures and Health) or (Expenditures and Indirect) or (Expenditures and "Out of Pocket") or (Expenditures and "Out-of Pocket") or (Expense and "Out-of-Pocket") or (Expenses and "Out-of-Pocket") or "Health Expenditure" or "Indirect Expenditure" or "Indirect Expenditures" or "Out of Pocket Costs" or "Out of Pocket Expenditures" or "Out of Pocket Expenses" or "Out of Pocket Payments" or "Out of Pocket Spending" or "Out-of Pocket Expenditure" or "Out-of Pocket Expenditures" or "Out-of-Pocket Cost" or "Out-of-Pocket Costs" or "Out-of-Pocket Expenditure" or "Out-of-Pocket Expenditures" or "Out-of-Pocket Expense" or "Out-of-Pocket Expenses" or "Out-of-Pocket Payment" or "Out-of-Pocket Payments" or "Out-of-Pocket Spending" or (payment and "Out-of-Pocket") or (Payments and "Out-of-Pocket") or (Spending and "Out-of-Pocket") or "Hospital Costs" or (Cost and Hospital) or (Costs and Hospital) or "Hospital Cost" or "Cost-Effectiveness Evaluation").mp. [mp=ti, ot, ab, sh, hw, kw, tx, ct, tn, dm, mf, dv, fx, dq, nm, kf, ox, px, rx, ui, sy] | 1673935 |
| 6 | 4 or 5 | 2016682 |
| 7 | 3 and 6 | 1052 |
| 8 | remove duplicates from 7  EBM Reviews - Cochrane Central Register of Controlled Trials <June 2019>  EBM Reviews - Cochrane Database of Systematic Reviews <2005 to July 24, 2019>  Embase <1974 to 2019 July 26>  Ovid MEDLINE(R) and Epub Ahead of Print, In-Process & Other Non-Indexed Citations, Daily and Versions(R) <1946 to July 26, 2019> | 874  28  103  653  90 |
| 9 | from 8 keep 1-28 | 28 |
| 10 | from 8 keep 29-131 | 103 |
| 11 | from 8 keep 132-784 | 653 |
| 12 | from 8 keep 785-874 | 90 |

| **Web of Science** | | |
| --- | --- | --- |
| **Step** | **Type** | **Results** |
| 1 | TS=("juvenile idiopathic arthritis" or "JIA" or "SJIA" or (Arthritis AND "Juvenile Chronic") or (Arthritis AND "Juvenile Enthesitis-Related") or (Arthritis AND "Juvenile Idiopathic") or (Arthritis AND "Juvenile Psoriatic") or (Arthritis AND "Juvenile Rheumatoid") or (Arthritis AND "Juvenile Systemic") or ("Chronic Arthritis" AND Juvenile) or ("Enthesitis Related Arthritis" AND Juvenile) or ("Enthesitis-Related Arthritis" AND Juvenile) or ("Idiopathic Arthritis" AND Juvenile) or "Juvenile Arthritis" or "Juvenile Chronic Arthritis" or "Juvenile Enthesitis-Related Arthritis" or "Juvenile Idiopathic Arthritis" or "Juvenile Oligoarthritis" or "Juvenile Onset Still Disease" or "Juvenile Onset Stills Disease" or "Juvenile Psoriatic Arthritis" or "Juvenile Rheumatoid Arthritis" or "Juvenile Systemic Arthritis" or "Juvenile-Onset Still Disease" or "Juvenile-Onset Still's Disease" or "Juvenile-Onset Stills Disease" or (ligoarthritis AND Juvenile) or (Polyarthritis AND Juvenile AND "Rheumatoid Factor Negative") or (Polyarthritis AND Juvenile AND "Rheumatoid Factor Positive") or ("Psoriatic Arthritis" AND Juvenile) OR ("Rheumatoid Arthritis" AND Juvenile) or ("Still Disease" AND "Juvenile Onset") or ("Still Disease" AND "Juvenile-Onset") or ("Still's Disease" AND "Juvenile Onset") or ("Still's Disease" AND "Juvenile-Onset") or ("Stills Disease" AND "Juvenile-Onset") or ("Systemic Arthritis" AND Juvenile))  *Índices=SCI-EXPANDED, ESCI Período de tiempo=Todos los años* | 15,945 |
| 2 | TS=(Cost or Costs or "Economic burden" or "related costs" or "direct healthcare costs" or "direct non-healthcare costs" or "loss of labor productivity" or "average annual costs" or (Costs and "Cost Analysis") or Affordabilities or Affordability or "Analyses cost" or "Cost-Minimization" or "Analysis cost" or "Cost Analyses" or "Cost Analysis" or "Cost Comparison" or "Cost Comparisons" or "Cost Measure" or "Cost Measures" or "Cost Minimization Analysis" or "Cost-Minimization Analyses" or "Cost-Minimization Analysis" or Pricing or "Cost Efficiency Analysis" or "Health Care Costs" or "Medical Care cost" or (Cost and Treatment) or (Costs and Health Care) or (Costs and Healthcare) or (Costs and "Medical Care") or (Costs and Treatment) or "Health Care Cost" or "Health Cost" or "Health Costs" or "Healthcare Cost" or "Healthcare Costs" or "Medical Care Cost" or "Medical Care Costs" or "Treatment Cost" or "Treatment Costs" or "Cost-Benefit Analysis" or "Cost Benefit" or "Cost-Benefit" or "Cost-Utility" or "Cost-Effectiveness" or "Cost-Utility" or (Benefits and Costs) or "Cost Benefit" or "Cost Effectiveness" or "Cost-Utility" or "Cost-Effectiveness" or "Cost-Utility" or "Economic Evaluation" or "Economic Evaluations" or (Evaluation and Economic) or (Evaluations and Economic) or "Health Expenditures" or "Out-of-Pocket" or "Direct Expenditure" or "Direct Expenditures" or "expenditures Indirect" or (expenditure and "Out-of Pocket") or Expenditures or (expenditures and Direct) or (Expenditures and Health) or (Expenditures and Indirect) or (Expenditures and "Out of Pocket") or (Expenditures and "Out-of Pocket") or (Expense and "Out-of-Pocket") or (Expenses and "Out-of-Pocket") or "Health Expenditure" or "Indirect Expenditure" or "Indirect Expenditures" or "Out of Pocket Costs" or "Out of Pocket Expenditures" or "Out of Pocket Expenses" or "Out of Pocket Payments" or "Out of Pocket Spending" or "Out-of Pocket Expenditure" or "Out-of Pocket Expenditures" or "Out-of-Pocket Cost" or "Out-of-Pocket Costs" or "Out-of-Pocket Expenditure" or "Out-of-Pocket Expenditures" or "Out-of-Pocket Expense" or "Out-of-Pocket Expenses" or "Out-of-Pocket Payment" or "Out-of-Pocket Payments" or "Out-of-Pocket Spending" or (payment and "Out-of-Pocket") or (Payments and "Out-of-Pocket") or (Spending and "Out-of-Pocket") or "Hospital Costs" or (Cost and Hospital) or (Costs and Hospital) or "Hospital Cost" or "Cost-Effectiveness Evaluation")  *Índices=SCI-EXPANDED, ESCI Período de tiempo=Todos los años* | 1,169,666 |
| 3 | #2 AND #1  *Índices=SCI-EXPANDED, ESCI Período de tiempo=Todos los años* | 266 |

| **SCOPUS** | | |
| --- | --- | --- |
| **Step** | **Type** | **Results** |
| 1 | TITLE-ABS-KEY ( ( ( "juvenile idiopathic arthritis"  OR  "JIA"  OR  "SJIA"  OR  ( arthritis  AND  "Juvenile Chronic" )  OR  ( arthritis  AND  "Juvenile Enthesitis-Related" )  OR  ( arthritis  AND  "Juvenile Idiopathic" )  OR  ( arthritis  AND  "Juvenile Psoriatic" )  OR  ( arthritis  AND  "Juvenile Rheumatoid" )  OR  ( arthritis  AND  "Juvenile Systemic" )  OR  ( "Chronic Arthritis"  AND  juvenile )  OR  ( "Enthesitis Related Arthritis"  AND  juvenile )  OR  ( "Enthesitis-Related Arthritis"  AND  juvenile )  OR  ( "Idiopathic Arthritis"  AND  juvenile )  OR  "Juvenile Arthritis"  OR  "Juvenile Chronic Arthritis"  OR  "Juvenile Enthesitis-Related Arthritis"  OR  "Juvenile Idiopathic Arthritis"  OR  "Juvenile Oligoarthritis"  OR  "Juvenile Onset Still Disease"  OR  "Juvenile Onset Stills Disease"  OR  "Juvenile Psoriatic Arthritis"  OR  "Juvenile Rheumatoid Arthritis"  OR  "Juvenile Systemic Arthritis"  OR  "Juvenile-Onset Still Disease"  OR  "Juvenile-Onset Still's Disease"  OR  "Juvenile-Onset Stills Disease"  OR  ( ligoarthritis  AND  juvenile )  OR  ( polyarthritis  AND  juvenile  AND  "Rheumatoid Factor Negative" )  OR  ( polyarthritis  AND  juvenile  AND  "Rheumatoid Factor Positive" )  OR  ( "Psoriatic Arthritis"  AND  juvenile )  OR  ( "Rheumatoid Arthritis"  AND  juvenile )  OR  ( "Still Disease"  AND  "Juvenile Onset" )  OR  ( "Still Disease"  AND  "Juvenile-Onset" )  OR  ( "Still's Disease"  AND  "Juvenile Onset" )  OR  ( "Still's Disease"  AND  "Juvenile-Onset" )  OR  ( "Stills Disease"  AND  "Juvenile-Onset" )  OR  ( "Systemic Arthritis"  AND  juvenile ) ) )  AND  ( ( cost  OR  costs  OR  "Economic burden"  OR  "related costs"  OR  "direct healthcare costs"  OR  "direct non-healthcare costs"  OR  "loss of labor productivity"  OR  "average annual costs"  OR  ( costs  AND  "Cost Analysis" )  OR  affordabilities  OR  affordability  OR  "Analyses cost"  OR  "Cost-Minimization"  OR  "Analysis cost"  OR  "Cost Analyses"  OR  "Cost Analysis"  OR  "Cost Comparison"  OR  "Cost Comparisons"  OR  "Cost Measure"  OR  "Cost Measures"  OR  "Cost Minimization Analysis"  OR  "Cost-Minimization Analyses"  OR  "Cost-Minimization Analysis"  OR  pricing  OR  "Cost Efficiency Analysis"  OR  "Health Care Costs"  OR  "Medical Care cost"  OR  ( cost  AND  treatment )  OR  ( costs  AND  health  AND  care )  OR  ( costs  AND  healthcare )  OR  ( costs  AND  "Medical Care" )  OR  ( costs  AND  treatment )  OR  "Health Care Cost"  OR  "Health Cost"  OR  "Health Costs"  OR  "Healthcare Cost"  OR  "Healthcare Costs"  OR  "Medical Care Cost"  OR  "Medical Care Costs"  OR  "Treatment Cost"  OR  "Treatment Costs"  OR  "Cost-Benefit Analysis"  OR  "Cost Benefit"  OR  "Cost-Benefit"  OR  "Cost-Utility"  OR  "Cost-Effectiveness"  OR  "Cost-Utility"  OR  ( benefits  AND  costs )  OR  "Cost Benefit"  OR  "Cost Effectiveness"  OR  "Cost-Utility"  OR  "Cost-Effectiveness"  OR  "Cost-Utility"  OR  "Economic Evaluation"  OR  "Economic Evaluations"  OR  ( evaluation  AND  economic )  OR  ( evaluations  AND  economic )  OR  "Health Expenditures"  OR  "Out-of-Pocket"  OR  "Direct Expenditure"  OR  "Direct Expenditures"  OR  "expenditures Indirect"  OR  ( expenditure  AND  "Out-of Pocket" )  OR  expenditures  OR  ( expenditures  AND  direct )  OR  ( expenditures  AND  health )  OR  ( expenditures  AND  indirect )  OR  ( expenditures  AND  "Out of Pocket" )  OR  ( expenditures  AND  "Out-of Pocket" )  OR  ( expense  AND  "Out-of-Pocket" )  OR  ( expenses  AND  "Out-of-Pocket" )  OR  "Health Expenditure"  OR  "Indirect Expenditure"  OR  "Indirect Expenditures"  OR  "Out of Pocket Costs"  OR  "Out of Pocket Expenditures"  OR  "Out of Pocket Expenses"  OR  "Out of Pocket Payments"  OR  "Out of Pocket Spending"  OR  "Out-of Pocket Expenditure"  OR  "Out-of Pocket Expenditures"  OR  "Out-of-Pocket Cost"  OR  "Out-of-Pocket Costs"  OR  "Out-of-Pocket Expenditure"  OR  "Out-of-Pocket Expenditures"  OR  "Out-of-Pocket Expense"  OR  "Out-of-Pocket Expenses"  OR  "Out-of-Pocket Payment"  OR  "Out-of-Pocket Payments"  OR  "Out-of-Pocket Spending"  OR  ( payment  AND  "Out-of-Pocket" )  OR  ( payments  AND  "Out-of-Pocket" )  OR  ( spending  AND  "Out-of-Pocket" )  OR  "Hospital Costs"  OR  ( cost  AND  hospital )  OR  ( costs  AND  hospital )  OR  "Hospital Cost"  OR  "Cost-Effectiveness Evaluation" ) ) )  AND  ( LIMIT-TO ( PUBYEAR ,  2020 )  OR  LIMIT-TO ( PUBYEAR ,  2019 )  OR  LIMIT-TO ( PUBYEAR ,  2018 )  OR  LIMIT-TO ( PUBYEAR ,  2017 )  OR  LIMIT-TO ( PUBYEAR ,  2016 )  OR  LIMIT-TO ( PUBYEAR ,  2015 )  OR  LIMIT-TO ( PUBYEAR ,  2014 )  OR  LIMIT-TO ( PUBYEAR ,  2013 )  OR  LIMIT-TO ( PUBYEAR ,  2012 )  OR  LIMIT-TO ( PUBYEAR ,  2011 )  OR  LIMIT-TO ( PUBYEAR ,  2010 )  OR  LIMIT-TO ( PUBYEAR ,  2009 )  OR  LIMIT-TO ( PUBYEAR ,  2008 )  OR  LIMIT-TO ( PUBYEAR ,  2007 )  OR  LIMIT-TO ( PUBYEAR ,  2006 )  OR  LIMIT-TO ( PUBYEAR ,  2005 )  OR  LIMIT-TO ( PUBYEAR ,  2004 )  OR  LIMIT-TO ( PUBYEAR ,  2003 )  OR  LIMIT-TO ( PUBYEAR ,  2002 )  OR  LIMIT-TO ( PUBYEAR ,  2001 )  OR  LIMIT-TO ( PUBYEAR ,  2000 ) ) | 548 |

**Summary March 11, 2021.**

| **Database** | **Before deduplication** | **After deduplication** |
| --- | --- | --- |
| MEDLINE | 8 | 4 |
| Embase | 67 | 62 |
| Web of Science | 30 | 16 |
| Scopus | 58 | 29 |
| CCRCT | 4 | 4 |
| CDSR | 5 | 5 |
| **TOTAL** | **172** | **120** |

**Examples**

| **MEDLINE** | | |
| --- | --- | --- |
| **Step** | **Type** | **Results** |
| 1 | exp juvenile rheumatoid arthritis/ or exp systemic juvenile idiopathic arthritis/ | 33103 |
| 2 | ("juvenile idiopathic arthritis" or "JIA" or "SJIA" or (Arthritis and "Juvenile Chronic") or (Arthritis and "Juvenile Enthesitis-Related") or (Arthritis and "Juvenile Idiopathic") or (Arthritis and "Juvenile Psoriatic") or (Arthritis and "Juvenile Rheumatoid") or (Arthritis and "Juvenile Systemic") or ("Chronic Arthritis" and Juvenile) or ("Enthesitis Related Arthritis" and Juvenile) or ("Enthesitis-Related Arthritis" and Juvenile) or ("Idiopathic Arthritis" and Juvenile) or "Juvenile Arthritis" or "Juvenile Chronic Arthritis" or "Juvenile Enthesitis-Related Arthritis" or "Juvenile Idiopathic Arthritis" or "Juvenile Oligoarthritis" or "Juvenile Onset Still Disease" or "Juvenile Onset Stills Disease" or "Juvenile Psoriatic Arthritis" or "Juvenile Rheumatoid Arthritis" or "Juvenile Systemic Arthritis" or "Juvenile-Onset Still Disease" or "Juvenile-Onset Still's Disease" or "Juvenile-Onset Stills Disease" or (ligoarthritis and Juvenile) or (Polyarthritis and Juvenile and "Rheumatoid Factor Negative") or (Polyarthritis and Juvenile and "Rheumatoid Factor Positive") or ("Psoriatic Arthritis" and Juvenile) or ("Rheumatoid Arthritis" and Juvenile) or ("Still Disease" and "Juvenile Onset") or ("Still Disease" and "Juvenile-Onset") or ("Still's Disease" and "Juvenile Onset") or ("Still's Disease" and "Juvenile-Onset") or ("Stills Disease" and "Juvenile-Onset") or ("Systemic Arthritis" and Juvenile)).mp. [mp=ti, ot, ab, sh, hw, kw, tx, ct, tn, dm, mf, dv, fx, dq, nm, kf, ox, px, rx, ui, sy] | 40030 |
| 3 | 1 or 2 | 42236 |
| 4 | exp "cost"/ or exp "cost of living"/ or exp "health care cost"/ | 986222 |
| 5 | (Cost or Costs or "Economic burden" or "related costs" or "direct healthcare costs" or "direct non-healthcare costs" or "loss of labor productivity" or "average annual costs" or (Costs and "Cost Analysis") or Affordabilities or Affordability or "Analyses cost" or "Cost-Minimization" or "Analysis cost" or "Cost Analyses" or "Cost Analysis" or "Cost Comparison" or "Cost Comparisons" or "Cost Measure" or "Cost Measures" or "Cost Minimization Analysis" or "Cost-Minimization Analyses" or "Cost-Minimization Analysis" or Pricing or "Cost Efficiency Analysis" or "Health Care Costs" or "Medical Care cost" or (Cost and Treatment) or (Costs and Health Care) or (Costs and Healthcare) or (Costs and "Medical Care") or (Costs and Treatment) or "Health Care Cost" or "Health Cost" or "Health Costs" or "Healthcare Cost" or "Healthcare Costs" or "Medical Care Cost" or "Medical Care Costs" or "Treatment Cost" or "Treatment Costs" or "Cost-Benefit Analysis" or "Cost Benefit" or "Cost-Benefit" or "Cost-Utility" or "Cost-Effectiveness" or "Cost-Utility" or (Benefits and Costs) or "Cost Benefit" or "Cost Effectiveness" or "Cost-Utility" or "Cost-Effectiveness" or "Cost-Utility" or "Economic Evaluation" or "Economic Evaluations" or (Evaluation and Economic) or (Evaluations and Economic) or "Health Expenditures" or "Out-of-Pocket" or "Direct Expenditure" or "Direct Expenditures" or "expenditures Indirect" or (expenditure and "Out-of Pocket") or Expenditures or (expenditures and Direct) or (Expenditures and Health) or (Expenditures and Indirect) or (Expenditures and "Out of Pocket") or (Expenditures and "Out-of Pocket") or (Expense and "Out-of-Pocket") or (Expenses and "Out-of-Pocket") or "Health Expenditure" or "Indirect Expenditure" or "Indirect Expenditures" or "Out of Pocket Costs" or "Out of Pocket Expenditures" or "Out of Pocket Expenses" or "Out of Pocket Payments" or "Out of Pocket Spending" or "Out-of Pocket Expenditure" or "Out-of Pocket Expenditures" or "Out-of-Pocket Cost" or "Out-of-Pocket Costs" or "Out-of-Pocket Expenditure" or "Out-of-Pocket Expenditures" or "Out-of-Pocket Expense" or "Out-of-Pocket Expenses" or "Out-of-Pocket Payment" or "Out-of-Pocket Payments" or "Out-of-Pocket Spending" or (payment and "Out-of-Pocket") or (Payments and "Out-of-Pocket") or (Spending and "Out-of-Pocket") or "Hospital Costs" or (Cost and Hospital) or (Costs and Hospital) or "Hospital Cost" or "Cost-Effectiveness Evaluation").mp. [mp=ti, ot, ab, sh, hw, kw, tx, ct, tn, dm, mf, dv, fx, dq, nm, kf, ox, px, rx, ui, sy] | 1902667 |
| 6 | 4 or 5 | 2260351 |
| 7 | 3 and 6 | 1223 |
| 8 | remove duplicates from 7 | 1007 |
| 9 | limit 8 to yr="2019 -Current" | 160 |
| 10 | from 9 keep 1-10 | 10 |
| 11 | from 9 keep 11-28 | 18 |
| 12 | from 9 keep 29-152 | 124 |
| 13 | from 9 keep 153-160 | 8 |

| **Web of Science** | | |
| --- | --- | --- |
| **Step** | **Type** | **Results** |
| 1 | TS=("juvenile idiopathic arthritis" or "JIA" or "SJIA" or (Arthritis AND "Juvenile Chronic") or (Arthritis AND "Juvenile Enthesitis-Related") or (Arthritis AND "Juvenile Idiopathic") or (Arthritis AND "Juvenile Psoriatic") or (Arthritis AND "Juvenile Rheumatoid") or (Arthritis AND "Juvenile Systemic") or ("Chronic Arthritis" AND Juvenile) or ("Enthesitis Related Arthritis" AND Juvenile) or ("Enthesitis-Related Arthritis" AND Juvenile) or ("Idiopathic Arthritis" AND Juvenile) or "Juvenile Arthritis" or "Juvenile Chronic Arthritis" or "Juvenile Enthesitis-Related Arthritis" or "Juvenile Idiopathic Arthritis" or "Juvenile Oligoarthritis" or "Juvenile Onset Still Disease" or "Juvenile Onset Stills Disease" or "Juvenile Psoriatic Arthritis" or "Juvenile Rheumatoid Arthritis" or "Juvenile Systemic Arthritis" or "Juvenile-Onset Still Disease" or "Juvenile-Onset Still's Disease" or "Juvenile-Onset Stills Disease" or (ligoarthritis AND Juvenile) or (Polyarthritis AND Juvenile AND "Rheumatoid Factor Negative") or (Polyarthritis AND Juvenile AND "Rheumatoid Factor Positive") or ("Psoriatic Arthritis" AND Juvenile) OR ("Rheumatoid Arthritis" AND Juvenile) or ("Still Disease" AND "Juvenile Onset") or ("Still Disease" AND "Juvenile-Onset") or ("Still's Disease" AND "Juvenile Onset") or ("Still's Disease" AND "Juvenile-Onset") or ("Stills Disease" AND "Juvenile-Onset") or ("Systemic Arthritis" AND Juvenile) )  *Índices=SCI-EXPANDED, ESCI Período de tiempo=2019-2021* | 1,995 |
| 2 | TS=(Cost or Costs or "Economic burden" or "related costs" or "direct healthcare costs" or "direct non-healthcare costs" or "loss of labor productivity" or "average annual costs" or (Costs and "Cost Analysis") or Affordabilities or Affordability or "Analyses cost" or "Cost-Minimization" or "Analysis cost" or "Cost Analyses" or "Cost Analysis" or "Cost Comparison" or "Cost Comparisons" or "Cost Measure" or "Cost Measures" or "Cost Minimization Analysis" or "Cost-Minimization Analyses" or "Cost-Minimization Analysis" or Pricing or "Cost Efficiency Analysis" or "Health Care Costs" or "Medical Care cost" or (Cost and Treatment) or (Costs and Health Care) or (Costs and Healthcare) or (Costs and "Medical Care") or (Costs and Treatment) or "Health Care Cost" or "Health Cost" or "Health Costs" or "Healthcare Cost" or "Healthcare Costs" or "Medical Care Cost" or "Medical Care Costs" or "Treatment Cost" or "Treatment Costs" or "Cost-Benefit Analysis" or "Cost Benefit" or "Cost-Benefit" or "Cost-Utility" or "Cost-Effectiveness" or "Cost-Utility" or (Benefits and Costs) or "Cost Benefit" or "Cost Effectiveness" or "Cost-Utility" or "Cost-Effectiveness" or "Cost-Utility" or "Economic Evaluation" or "Economic Evaluations" or (Evaluation and Economic) or (Evaluations and Economic) or "Health Expenditures" or "Out-of-Pocket" or "Direct Expenditure" or "Direct Expenditures" or "expenditures Indirect" or (expenditure and "Out-of Pocket") or Expenditures or (expenditures and Direct) or (Expenditures and Health) or (Expenditures and Indirect) or (Expenditures and "Out of Pocket") or (Expenditures and "Out-of Pocket") or (Expense and "Out-of-Pocket") or (Expenses and "Out-of-Pocket") or "Health Expenditure" or "Indirect Expenditure" or "Indirect Expenditures" or "Out of Pocket Costs" or "Out of Pocket Expenditures" or "Out of Pocket Expenses" or "Out of Pocket Payments" or "Out of Pocket Spending" or "Out-of Pocket Expenditure" or "Out-of Pocket Expenditures" or "Out-of-Pocket Cost" or "Out-of-Pocket Costs" or "Out-of-Pocket Expenditure" or "Out-of-Pocket Expenditures" or "Out-of-Pocket Expense" or "Out-of-Pocket Expenses" or "Out-of-Pocket Payment" or "Out-of-Pocket Payments" or "Out-of-Pocket Spending" or (payment and "Out-of-Pocket") or (Payments and "Out-of-Pocket") or (Spending and "Out-of-Pocket") or "Hospital Costs" or (Cost and Hospital) or (Costs and Hospital) or "Hospital Cost" or "Cost-Effectiveness Evaluation")  *Índices=SCI-EXPANDED, ESCI Período de tiempo=2019-2021* | 295,528 |
| 3 | #2 AND #1  *Índices=SCI-EXPANDED, ESCI Período de tiempo=2019-2021* | 30 |

| **SCOPUS** | | |
| --- | --- | --- |
| **Step** | **Type** | **Results** |
| 1 | TITLE-ABS-KEY ( ( ( "juvenile idiopathic arthritis"  OR  "JIA"  OR  "SJIA"  OR  ( arthritis  AND  "Juvenile Chronic" )  OR  ( arthritis  AND  "Juvenile Enthesitis-Related" )  OR  ( arthritis  AND  "Juvenile Idiopathic" )  OR  ( arthritis  AND  "Juvenile Psoriatic" )  OR  ( arthritis  AND  "Juvenile Rheumatoid" )  OR  ( arthritis  AND  "Juvenile Systemic" )  OR  ( "Chronic Arthritis"  AND  juvenile )  OR  ( "Enthesitis Related Arthritis"  AND  juvenile )  OR  ( "Enthesitis-Related Arthritis"  AND  juvenile )  OR  ( "Idiopathic Arthritis"  AND  juvenile )  OR  "Juvenile Arthritis"  OR  "Juvenile Chronic Arthritis"  OR  "Juvenile Enthesitis-Related Arthritis"  OR  "Juvenile Idiopathic Arthritis"  OR  "Juvenile Oligoarthritis"  OR  "Juvenile Onset Still Disease"  OR  "Juvenile Onset Stills Disease"  OR  "Juvenile Psoriatic Arthritis"  OR  "Juvenile Rheumatoid Arthritis"  OR  "Juvenile Systemic Arthritis"  OR  "Juvenile-Onset Still Disease"  OR  "Juvenile-Onset Still's Disease"  OR  "Juvenile-Onset Stills Disease"  OR  ( ligoarthritis  AND  juvenile )  OR  ( polyarthritis  AND  juvenile  AND  "Rheumatoid Factor Negative" )  OR  ( polyarthritis  AND  juvenile  AND  "Rheumatoid Factor Positive" )  OR  ( "Psoriatic Arthritis"  AND  juvenile )  OR  ( "Rheumatoid Arthritis"  AND  juvenile )  OR  ( "Still Disease"  AND  "Juvenile Onset" )  OR  ( "Still Disease"  AND  "Juvenile-Onset" )  OR  ( "Still's Disease"  AND  "Juvenile Onset" )  OR  ( "Still's Disease"  AND  "Juvenile-Onset" )  OR  ( "Stills Disease"  AND  "Juvenile-Onset" )  OR  ( "Systemic Arthritis"  AND  juvenile ) ) )  AND  ( ( cost  OR  costs  OR  "Economic burden"  OR  "related costs"  OR  "direct healthcare costs"  OR  "direct non-healthcare costs"  OR  "loss of labor productivity"  OR  "average annual costs"  OR  ( costs  AND  "Cost Analysis" )  OR  affordabilities  OR  affordability  OR  "Analyses cost"  OR  "Cost-Minimization"  OR  "Analysis cost"  OR  "Cost Analyses"  OR  "Cost Analysis"  OR  "Cost Comparison"  OR  "Cost Comparisons"  OR  "Cost Measure"  OR  "Cost Measures"  OR  "Cost Minimization Analysis"  OR  "Cost-Minimization Analyses"  OR  "Cost-Minimization Analysis"  OR  pricing  OR  "Cost Efficiency Analysis"  OR  "Health Care Costs"  OR  "Medical Care cost"  OR  ( cost  AND  treatment )  OR  ( costs  AND  health  AND  care )  OR  ( costs  AND  healthcare )  OR  ( costs  AND  "Medical Care" )  OR  ( costs  AND  treatment )  OR  "Health Care Cost"  OR  "Health Cost"  OR  "Health Costs"  OR  "Healthcare Cost"  OR  "Healthcare Costs"  OR  "Medical Care Cost"  OR  "Medical Care Costs"  OR  "Treatment Cost"  OR  "Treatment Costs"  OR  "Cost-Benefit Analysis"  OR  "Cost Benefit"  OR  "Cost-Benefit"  OR  "Cost-Utility"  OR  "Cost-Effectiveness"  OR  "Cost-Utility"  OR  ( benefits  AND  costs )  OR  "Cost Benefit"  OR  "Cost Effectiveness"  OR  "Cost-Utility"  OR  "Cost-Effectiveness"  OR  "Cost-Utility"  OR  "Economic Evaluation"  OR  "Economic Evaluations"  OR  ( evaluation  AND  economic )  OR  ( evaluations  AND  economic )  OR  "Health Expenditures"  OR  "Out-of-Pocket"  OR  "Direct Expenditure"  OR  "Direct Expenditures"  OR  "expenditures Indirect"  OR  ( expenditure  AND  "Out-of Pocket" )  OR  expenditures  OR  ( expenditures  AND  direct )  OR  ( expenditures  AND  health )  OR  ( expenditures  AND  indirect )  OR  ( expenditures  AND  "Out of Pocket" )  OR  ( expenditures  AND  "Out-of Pocket" )  OR  ( expense  AND  "Out-of-Pocket" )  OR  ( expenses  AND  "Out-of-Pocket" )  OR  "Health Expenditure"  OR  "Indirect Expenditure"  OR  "Indirect Expenditures"  OR  "Out of Pocket Costs"  OR  "Out of Pocket Expenditures"  OR  "Out of Pocket Expenses"  OR  "Out of Pocket Payments"  OR  "Out of Pocket Spending"  OR  "Out-of Pocket Expenditure"  OR  "Out-of Pocket Expenditures"  OR  "Out-of-Pocket Cost"  OR  "Out-of-Pocket Costs"  OR  "Out-of-Pocket Expenditure"  OR  "Out-of-Pocket Expenditures"  OR  "Out-of-Pocket Expense"  OR  "Out-of-Pocket Expenses"  OR  "Out-of-Pocket Payment"  OR  "Out-of-Pocket Payments"  OR  "Out-of-Pocket Spending"  OR  ( payment  AND  "Out-of-Pocket" )  OR  ( payments  AND  "Out-of-Pocket" )  OR  ( spending  AND  "Out-of-Pocket" )  OR  "Hospital Costs"  OR  ( cost  AND  hospital )  OR  ( costs  AND  hospital )  OR  "Hospital Cost"  OR  "Cost-Effectiveness Evaluation" ) ) )  AND  ( LIMIT-TO ( PUBYEAR ,  2021 )  OR  LIMIT-TO ( PUBYEAR ,  2020 )  OR  LIMIT-TO ( PUBYEAR ,  2019 ) ) | 58 |
