# Supplementary material for: Economic impact of Juvenile Idiopathic Arthritis: a systematic review
Source: Pediatr Rheumatol Online J. 2021 Oct 9;19:152. doi: 10.1186/s12969-021-00641-y (PMC8502332; doi:10.1186/s12969-021-00641-y)
Supplement: Supplementary file 2 — Additional file 2. Supplementary tables. [file 12969_2021_641_MOESM2_ESM.docx]

**SUPPLEMENTARY TABLES**

Supplementary Table 1. Distribution of patients by category of JIA.

| Article | Oligoarthritis | Polyarthritis^a^ | ERA | Systemic | Psoriatic | Undifferentiated | Total |
| --- | --- | --- | --- | --- | --- | --- | --- |
| Angelis, 2016 | 6 | 6 | 1 | 7 | 1 | 2 | 23 |
| Bernatsky, 2007 | 67 | 31 | 15 | 14 | 16 | 12 | 155 |
| Ens, 2013 | 21 | 9 | 8 | 7 | 7 | 2 | 54 |
| Minden, 2004 | 85 | 30 | 33 | 30 | 3 | 34 | 215 |
| Haapasaari, 2004 | 6 | 22 |  | 3 |  |  | 31 |
| Yucel, 2012 | 30 | 45 | 6 | 13 | 6 |  | 100 |
| Minden, 2009 | 178 | 60 | 58 | 21 | 25 | 27 | 369 |
| Prince, 2011 | 11 | 22 | 2 | 11 | 3 |  | 49 |
| Shenoi, 2018 |  |  |  | 61 |  |  | 61 |
| Thornton, 2008 | 156 | 50 | 24 | 17 | 17 | 12 | 276 |
| Kip, 2020 | 364 | 175 | 59 | 57 | 29 | 7 | 691 |
| Thakral, 2020 | 97 |  |  |  |  |  | 97 |
| Total | 1021 | 450 | 206 | 241 | 107 | 96 | 2121 |
| Percentage | 48.1 | 21.2 | 9.7 | 11.4 | 5.0 | 4.5 | 100 |

ERA: Enthesitis-related arthritis.
^a^ Patients with positive and negative rheumatoid factor are grouped together.

Supplementary Table 2. Direct Health Care costs by category of JIA.

|  | Rheumatology Medical appointments^a^ | Other Doctors Medical appointments^a^ | Medications^a^ | | Transportation^a^ |
| --- | --- | --- | --- | --- | --- |
| Article | Thornton, 2008(1) | Thornton, 2008(1) | Thornton, 2008(1) | Kip, 2020(2) | Shenoi, 2018(3) |
| Original currency | GBP 2008 | GBP 2008 | GBP 2008 | EUR 2019 | USD 2016 |
| Oligoarthritis | 1,608 | 852 | 336 | 1,299 |  |
| Polyarthritis | 1,946 | 1,192 | 380 | 3,496 |  |
| ERA | 1,979 | 833 | 590 | 3,019 |  |
| Psoriasic | 1,314 | 821 | 343 | 3,823 |  |
| Systemic | 2,502 | 611 | 744 | 5,548 | 1,014 |
| Undifferentiated | 2,249 | 560 | 299 | 29 |  |

ERA: Enthesitis-related arthritis. USD: United States dollar. EUR: Euro. GBP: Great Britain pound.

^a^ Adjusted to inflation and converted to US Dollar. Based on exchange rate on December 31, 2019.

Supplementary Table 3. Total, direct, and indirect costs by country reported by Kuhlmann, 2016(4) (EUR 2012).

| Country | Total Costs^a^ | Direct Health Care Costs^a^ | Direct non-Health Care Costs^a^ | Indirect Costs^a^ |
| --- | --- | --- | --- | --- |
| Bulgary | 4,050 | 3,921 | 129 |  |
| France | 26,878 | 15,730 | 11,149 |  |
| Germany | 39,143 | 22,967 | 16,176 | 129 |
| Italy | 40,708 | 29,571 | 11,137 |  |
| Sweden | 51,578 | 31,461 | 20,166 | 147 |
| United Kingdom | 44,832 | 20,618 | 11,828 | 12,385 |

^a^ Adjusted to inflation and converted to US Dollar. Based on exchange rate on December 31, 2019.

Supplementary Table 4. Quality of Health Economic Studies (QHES) scores.

| **Criteria** | **Angelis, 2016**(5) | **Hughes, 2018**(6) | **Kuhlmann, 2016**(4) | **Minden, 2004(7)** | **Minden, 2009**(8) | **Khatun, 2021**(9) | **Kip, 2020**(2) | **Thakral, 2020**(10) | **Prince, 2011**(11) | **Shenoi, 2018**(3) | **Thornton, 2008**(1) |
| --- | --- | --- | --- | --- | --- | --- | --- | --- | --- | --- | --- |
| **1** | + | + | + | + | + | + | + | + | + | + | + |
| **2** | + | + | + | + | + | + | + | + | + | + | + |
| **3** | + | + | + | + | + | + | + | + | + | + | + |
| **4** | + | + | + | + | + | + | * | + | * | + | * |
| **5** | + | + | + | + | * | + | + | * | + | * | + |
| **6** | * | * | * | * | * | + | + | * | * | + | * |
| **7** | + | + | + | + | + | + | + | + | + | * | + |
| **8** | * | * | * | * | + | * | * | * | + | * | * |
| **9** | + | + | + | + | + | + | + | + | + | + | + |
| **10** | + | + | + | + | + | + | + | + | + | + | * |
| **11** | + | + | + | + | + | + | + | + | + | * | + |
| **12** | + | + | + | + | + | + | + | + | + | + | + |
| **13** | + | + | + | + | + | + | + | + | + | * | + |
| **14** | + | + | + | + | + | + | + | + | + | + | * |
| **15** | + | + | + | + | + | + | + | + | + | + | + |
| **16** | + | + | + | * | + | * | * | * | * | * | + |
| Total | 87/100 | 87/100 | 87/100 | 84/100 | 85/100 | 90/100 | 89/100 | 75/100 | 90/100 | 62/100 | 78/100 |

* Study met the criterion.

+ Study did not meet the criterion.

‡ Not applicable.

Note: Sixteen criteria of the Quality of Health Economic Studies (QHES) Instrument: 1. Was the study objective presented in a clear, specific, and measurable manner? (7 points); 2. Were the perspective of the analysis (societal, third-party payer, etc.) and reasons for its selection stated? (4 points); 3. Were variable estimates used in the analysis from the best available source (i.e., randomized control trial - best, expert opinion - worst)? (8 points); 4. If estimates came from a subgroup analysis, were the groups prespecified at the beginning of the study? (1 point); 5. Was uncertainty handled by (1) statistical analysis to address random events, (2) sensitivity analysis to cover a range of assumptions? (9 points); 6. Was incremental analysis performed between alternatives for resources and costs? (6 points); 7. Was the methodology for data abstraction (including the value of health states and other benefits) stated? (5 points); 8. Did the analytic horizon allow time for all relevant and important outcomes? Were benefits and costs that went beyond 1 year discounted (3% to 5%) and justification given for the discount rate? (7 points); 9. Was the measurement of costs appropriate and the methodology for the estimation of quantities and unit costs clearly described? (8 points); 10. Were the primary outcome measure(s) for the economic evaluation clearly stated and, were the major short-term, long-term and negative outcomes included? (6 points); 11. Were the health outcomes measures/scales valid and reliable? If previously tested valid and reliable measures were not available, was justification given for the measures/scales used? (7 points); 12. Were the economic model (including structure), study methods and analysis, and the components of the numerator and denominator displayed in a clear, transparent manner? (8 points); 13. Were the choice of economic model, main assumptions, and limitations of the study stated and justified? (7 points); 14. Did the author(s) explicitly discuss direction and magnitude of potential biases? (6 points); 15. Were the conclusions/recommendations of the study justified and based on the study results? (8 points); 16. Was there a statement disclosing the source of funding for the study? (3 points). The summatory of the criteria met by the study was considered as good quality when scored 75 or higher, otherwise, was considered as low quality.

Supplementary Table 5. NIH Quality Assessment Tool for Observational Cohort and Cross-Sectional Studies scores.

| **Criteria** | **Bernatsky, 2007**(12) | **Mars, 2019**(13) | **Mars, 2019**(14) |
| --- | --- | --- | --- |
| **1** | + | + | + |
| **2** | + | + | + |
| **3** | + | + | + |
| **4** | + | + | + |
| **5** | + | + | + |
| **6** | + | + | * |
| **7** | + | + | + |
| **8** | * | * | * |
| **9** | + | + | + |
| **10** | * | * | * |
| **11** | + | + | + |
| **12** | * | * | * |
| **13** | + | + | + |
| **14** | + | + | + |
|  | Low risk | Low risk | Low risk |

* Study met the criterion.

+ Study did not meet the criterion.

‡ Not applicable.

Note: Fourteen criteria of the NIH Quality Assessment Tool for Observational Cohort and Cross-Sectional Studies. 1. Was the research question or objective in this paper clearly stated? 2. Was the study population clearly specified and defined? 3. Was the participation rate of eligible persons at least 50%? 4. Were all the subjects selected or recruited from the same or similar populations (including the same time period)? Were inclusion and exclusion criteria for being in the study prespecified and applied uniformly to all participants? 5. Was a sample size justification, power description, or variance and effect estimates provided? 6. For the analyses in this paper, were the exposure(s) of interest measured prior to the outcome(s) being measured? 7. Was the timeframe sufficient so that one could reasonably expect to see an association between exposure and outcome if it existed? 8. For exposures that can vary in amount or level, did the study examine different levels of the exposure as related to the outcome (e.g., categories of exposure, or exposure measured as continuous variable)? 9. Were the exposure measures (independent variables) clearly defined, valid, reliable, and implemented consistently across all study participants? 10. Was the exposure(s) assessed more than once over time? 11. Were the outcome measures (dependent variables) clearly defined, valid, reliable, and implemented consistently across all study participants? 12. Were the outcome assessors blinded to the exposure status of participants? 13. Was loss to follow-up after baseline 20% or less? 14. Were key potential confounding variables measured and adjusted statistically for their impact on the relationship between exposure(s) and outcome(s)?

Supplementary Table 6. Newcastle-Ottawa Quality Assessment Form for Cohort Studies scores.

| **Criteria** | **Ens, 2013**(15) | **Haapasaari, 2004**(16) | **Yucel, 2012**(17) | **Marshall, 2019**(18) |
| --- | --- | --- | --- | --- |
| **1** | * | * | * | * |
| **2** | * | * | * | * |
| **3** | * | * | * | * |
| **4** | + | + | + | + |
| **Selection Stars** | 3/4 | 3/4 | 3/4 | 3/4 |
| **1** | * | * | * | * |
| **2** | + | + | + | + |
| **Comparability Stars** | 1/2 | 1/2 | 1/2 | 1/2 |
| **1** | * | * | * | * |
| **2** | * | * | * | * |
| **3** | ‡ | ‡ | ‡ | ‡ |
| **Outcome Stars** | 2/3 | 2/3 | 2/3 | 2/3 |
| **Total** | 6 good quality | 6 good quality | 6 good quality | 6 good quality |

* Study met the criterion (give a star).

+ Study did not meet the criterion.

‡ Not applicable.

Note: Eight criteria of the Newcastle-Ottawa Quality Assessment Form for Cohort Studies divided in three domains (Selection/Comparability/Outcome). **Selection:** 1) Representativeness of the exposed cohort 2) Selection of the non-exposed cohort 3) Ascertainment of exposure 4) Demonstration that outcome of interest was not present at start of study. **Comparability:** 1) Comparability of cohorts on the basis of the design or analysis controlled for confounders. **Outcome:** 1) Assessment of outcome 2) Was follow-up long enough for outcomes to occur 3) Adequacy of follow-up of cohorts.

Final score:

- Good quality: 3 or 4 stars in selection domain AND 1 or 2 stars in comparability domain AND 2 or 3 stars in outcome/exposure domain.
- Fair quality: 2 stars in selection domain AND 1 or 2 stars in comparability domain AND 2 or 3 stars in outcome/exposure domain.
- Poor quality: 0 or 1 star in selection domain OR 0 stars in comparability domain OR 0 or 1 stars in outcome/exposure domain.

**REFERENCES**

1. Thornton J, Lunt M, Ashcroft DM, Baildam E, Foster H, Davidson J, et al. Costing juvenile idiopathic arthritis: examining patient-based costs during the first year after diagnosis. Rheumatology. el 25 de abril de 2008;47(7):985–90.

2. Kip MMA, de Roock S, Currie G, Marshall DA, Grazziotin LR, Twilt M, et al. Costs of medication use among patients with juvenile idiopathic arthritis in the Dutch healthcare system. Expert Rev Pharmacoecon Outcomes Res. el 29 de diciembre de 2020;1–10.

3. Shenoi S, Horneff G, Cidon M, Ramanan AV, Kimura Y, Quartier P, et al. The burden of systemic juvenile idiopathic arthritis for patients and caregivers: an international survey and retrospective chart review. Clin Exp Rheumatol. octubre de 2018;36(5):920–8.

4. Kuhlmann A, Schmidt T, Treskova M, López-Bastida J, Linertová R, Oliva-Moreno J, et al. Social/economic costs and health-related quality of life in patients with juvenile idiopathic arthritis in Europe. Eur J Health Econ HEPAC Health Econ Prev Care. abril de 2016;17 Suppl 1:79–87.

5. Angelis A, Kanavos P, López-Bastida J, Linertová R, Serrano-Aguilar P, BURQOL-RD Research Network. Socioeconomic costs and health-related quality of life in juvenile idiopathic arthritis: a cost-of-illness study in the United Kingdom. BMC Musculoskelet Disord. diciembre de 2016;17(1):321.

6. Hughes DA, Culeddu G, Plumpton CO, Wood E, Dick AD, Jones AP, et al. Cost-Effectiveness Analysis of Adalimumab for the Treatment of Uveitis Associated with Juvenile Idiopathic Arthritis. Ophthalmology. marzo de 2019;126(3):415–24.

7. Minden K. Burden and cost of illness in patients with juvenile idiopathic arthritis. Ann Rheum Dis. el 1 de julio de 2004;63(7):836–42.

8. Minden K, Niewerth M, Listing J, Möbius D, Thon A, Ganser G, et al. The economic burden of juvenile idiopathic arthritis-results from the German paediatric rheumatologic database. Clin Exp Rheumatol. octubre de 2009;27(5):863–9.

9. Khatun M, Datta D, Hazra A, Ghosh P, Selim MB, Mondal R. Economic Burden of Juvenile Idiopathic Arthritis in India. Indian Pediatr. el 15 de enero de 2021;58(1):38–40.

10. Thakral A, Pinto D, Miller M, Curran ML, Klein-Gitelman M, French DD. Direct Healthcare Costs Associated with Oligoarticular Juvenile Idiopathic Arthritis at a Single Center. Int J Rheumatol. el 1 de septiembre de 2020;2020:1–5.

11. Prince FHM, de Bekker-Grob EW, Twilt M, van Rossum MAJ, Hoppenreijs EPAH, ten Cate R, et al. An analysis of the costs and treatment success of etanercept in juvenile idiopathic arthritis: results from the Dutch Arthritis and Biologicals in Children register. Rheumatology. el 1 de junio de 2011;50(6):1131–6.

12. Bernatsky S, Duffy C, Malleson P, Feldman DE, St. Pierre Y, Clarke AE. Economic impact of juvenile idiopathic arthritis. Arthritis Rheum. el 15 de febrero de 2007;57(1):44–8.

13. Mars NJ, Kerola AM, Kauppi MJ, Pirinen M, Elonheimo O, Sokka-Isler T. Healthcare costs and outcomes in adult patients with juvenile idiopathic arthritis: a population-based study. Scand J Rheumatol. marzo de 2019;48(2):114–20.

14. Mars N, Kerola A, Kauppi M, Pirinen M, Elonheimo O, Sokka-Isler T. Patients with rheumatic diseases share similar patterns of healthcare resource utilization. Scand J Rheumatol. el 4 de julio de 2019;48(4):300–7.

15. Ens A, Lang B, Ramsey S, Stringer E, Huber AM. The financial burden of juvenile idiopathic arthritis: a Nova Scotia experience. Pediatr Rheumatol. 2013;11(1):24.

16. Haapasaari J, Kautiainen HJ, Isomäki HA, Hakala M. Etanercept does not essentially increase the total costs of the treatment of refractory juvenile idiopathic arthritis. J Rheumatol. noviembre de 2004;31(11):2286–9.

17. Yucel IK, Seyahi E, Kasapcopur O, Arisoy N. Economic impact of juvenile idiopathic arthritis and familial Mediterranean fever. Rheumatol Int. julio de 2012;32(7):1955–62.

18. Marshall A, Gupta K, Pazirandeh M, Bonafede M, McMorrow D. Treatment patterns and economic outcomes in patients with juvenile idiopathic arthritis. Clin Outcomes Res. mayo de 2019;Volume 11:361–71.
